# Identification and interrogation of combinatorial histone modifications

**DOI:** 10.3389/fgene.2013.00264

**Published:** 2013-12-20

**Authors:** Kelly R. Karch, Jamie E. DeNizio, Ben E. Black, Benjamin A. Garcia

**Affiliations:** Epigenetics Program, Department of Biochemistry and Biophysics, Perelman School of Medicine, University of PennsylvaniaPhiladelphia, PA, USA

**Keywords:** mass spectrometry, histone, histone code, post-translational modification, proteomics, chromatin, histone variants, deuterium exchange

## Abstract

Histone proteins are dynamically modified to mediate a variety of cellular processes including gene transcription, DNA damage repair, and apoptosis. Regulation of these processes occurs through the recruitment of non-histone proteins to chromatin by specific combinations of histone post-translational modifications (PTMs). Mass spectrometry has emerged as an essential tool to discover and quantify histone PTMs both within and between samples in an unbiased manner. Developments in mass spectrometry that allow for characterization of large histone peptides or intact protein has made it possible to determine which modifications occur simultaneously on a single histone polypeptide. A variety of techniques from biochemistry, biophysics, and chemical biology have been employed to determine the biological relevance of discovered combinatorial codes. This review first describes advancements in the field of mass spectrometry that have facilitated histone PTM analysis and then covers notable approaches to probe the biological relevance of these modifications in their nucleosomal context.

## INTRODUCTION

Nucleosomes, the basic repeating unit of chromatin, consist of ~147 bp DNA wound around a histone core containing two copies of the histone proteins H2A, H2B, H3, and H4 (Luger et al., 1997). Histones undergo dynamic post-translational modifications (PTMs) on specific residues, most of which are contained on the flexible N-terminal tail that protrudes from the nucleosomal surface (Cosgrove, 2007). It has been hypothesized that PTMs may form a “histone code” in which particular marks or combinations of marks elicit a specific physiological response by regulating chromatin structure (Jenuwein and Allis, 2001). PTMs may perform these tasks by directly altering the chemical environment of the surrounding chromatin or through the action of other proteins that bind to these marks, termed readers. Readers may contain or recruit effector proteins, forming a signaling scaffold to alter chromatin function and consequently mediate processes such as gene expression, apoptosis, and DNA damage repair (Jenuwein and Allis, 2001).

Histone modification analysis has traditionally been conducted using site-specific antibody-based methods such as western blots, chromatin immunoprecipitation, DNA microarrays, and deep sequencing (Britton et al., 2011). While these types of methods are useful in studying histone PTMs, there are several drawbacks. Firstly, the development of modification-specific antibodies is difficult, costly, and challenging to validate. One major concern is cross-reaction with similar modifications on the same or a different histone protein. Epitope occlusion, where nearby PTMs block antibody binding, can also be a major issue, especially on highly modified histones. Furthermore, antibody-based methods require *a priori* knowledge of particular marks, making discovery of novel marks difficult. Mass spectrometry (MS), on the other hand, provides an unbiased, highly quantitative, and comparative approach for studying histone modifications (Zee et al., 2011). One of the greatest advantages of MS is the ability to identify novel PTMs and measure the co-occurrence of modifications on the same peptide. As such, MS has emerged as one of the most powerful tools for histone modification analysis. The first half of this review discusses modern MS techniques used to characterize histones and identify combinatorial PTM codes, as briefly summarized in **Table 1**.

**Table 1:** Comparison of MS techniques.

	Scope	Advantages	Disadvantages
Bottom up	Small peptide fragments	-Best sensitivity	-Lose connectivity of most PTMs
		-Easiest analysis	-Generally paired with CID
			-Labile PTMs lost
			-Non-random backbone cleavage
Middle down	Medium peptide fragments (~50 AA)	-Better connectivity than bottom up peptides	-Lose connectivity of some PTMs
		-Better sensitivity than top-down	-Paired with ETD
			-Retain labile PTMs
			-Even backbone cleavage
Top down	Entire proteins	-Complete connectivity of PTMs	-Difficult data analysis
			-Worst sensitivity
			-Paired with ETD
			-Retain labile PTMs
			-Even backbone cleavage

As MS and other techniques identify novel PTM profiles, questions about the biological relevance of these profiles emerge. Many studies aim to identify the “writer” and “eraser” enzymes responsible for the addition and removal of the PTMs, respectively, to gain a better understanding of how these marks are dynamically regulated. Identification of the reader proteins is vital for understanding how combinatorial codes lead to a specific physiological response. Investigators have also aimed to characterize the impact of a specific combination of PTMs on chromatin organization and nucleosomal structure. Many of these types of studies are facilitated by chemical biology techniques that allow for the synthesis of homogenous pools of histones containing identical PTM combinations (Chatterjee and Muir, 2010; Fierz and Muir, 2012). The second half of this review focuses on useful techniques that can be used to characterize histone function.

## MASS SPECTROMETRY FOR HISTONE ANALYSIS

### BOTTOM-UP MASS SPECTROMETRY

**FIGURE 1:**
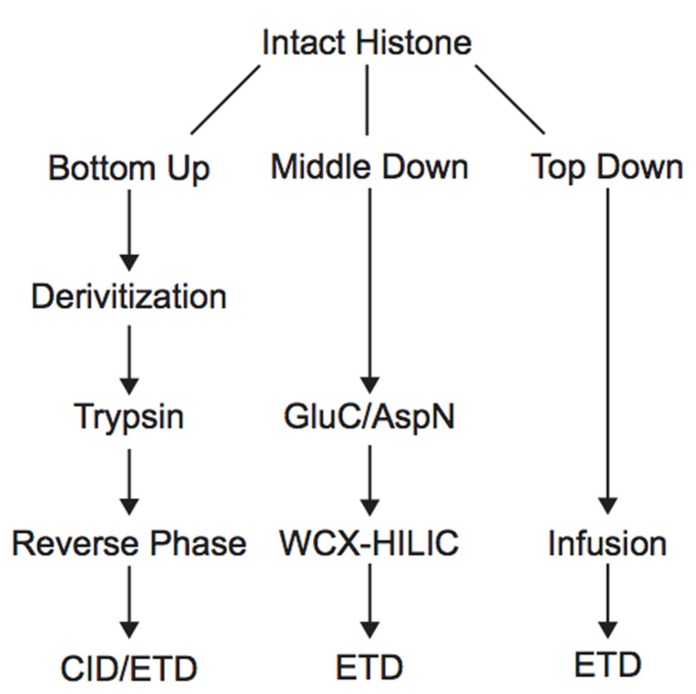
**Workflow for bottom-up, middle-down, and top-down mass spectrometry experiments**.

Histone PTM analysis via MS can be completed in several ways (**Figure 1**). Bottom-up MS involves digesting histones to generate small peptide fragments followed by on- or off-line chromatographic separation and MS analysis for sequencing and quantification (Chait, 2006). Bottom-up peptides are typically separated via reverse-phase high performance liquid chromatography (RP-HPLC), which uses a hydrophobic stationary phase and polar mobile phase. Many bottom-up histone studies employ trypsin for digestion due to its high efficiency and specificity for lysines and arginines, allowing for the generation of highly reproducible peptides (Olsen et al., 2004). Trypsin cleavage can be problematic for histone PTM analysis, however, because histone tails contain many lysines and arginines, resulting in many very small peptides that do not retain well on RP columns. Furthermore, histones contain several adjacent lysines and arginines that can lead to trypsin missed-cleavage events as the enzyme will only cleave after one of the adjacent residues at random. Trypsin also fails to cleave peptides at modified lysines or arginines due to charge neutralization. Missed-cleavage events can result in a non-homogenous pool of peptides in which the same PTM site exists on several different peptides, making quantitation of these marks extremely difficult.

Derivatization of histone samples prior to trypsin digestion can circumvent these issues. For example, the Smith group designed a method that employed deuterated acetic anhydride to acetylate all lysines, blocking them from tryptic digestion. Trypsin will therefore only cleave C-terminal to arginine residues, reducing the number of missed-cleavage events. Furthermore, using deuterated acetic acid allows for the differentiation of endogenously acetylated residues from chemically acetylated residues (Δm = 3.018 Da). Using this method, Smith et al. (2002, 2003) were able to generate a homogenous pool of the H4 4–17 peptide and compare the acetylation level of each lysine between wild type and mutant yeast cells displaying increased telomeric silencing. They discovered a reduction in the level of acetylation of H3K12 in the mutant cells, highlighting this PTM as an important regulator of gene silencing. Another similar derivatization technique uses propionic anhydride to propionylate unmodified and monomethylated lysines to block them from trypsin cleavage. This modification is less likely to be confused with endogenous acetylation or trimethylation because it imparts a much greater mass shift (Δm = 14 Da) than a deuterated acetyl group. The propionyl group also increases the hydrophobicity of the peptide, allowing for enhanced chromatographic resolution via RP (Garcia et al., 2007a). Recently, Chen et al. (2011) used this method to generate a reliable pool of tryptic peptides to determine if lysine methylation is symmetrical between the two histone copies within a nucleosome for 18 major lysine methylation states. The authors constructed nucleosomes containing one wild type and one unmethylatable histone, and compared the level of lysine methylation on the wild type histone from this pair to wild type histone paired with wild type. Results demonstrated that lysine methylation levels were very similar between the two types of pairs, indicating that methylation states of histone copies within the nucleosome do not need to be symmetrical, although exceptions could exist (Chen et al., 2011). A later study employed site-specific antibodies and bottom-up MS to directly demonstrate that both symmetrically and asymmetrically modified nucleosomes exist. The study also demonstrated that histone-modifying proteins can recognize the symmetry of specific marks within the nucleosome (Voigt et al., 2012).

**FIGURE 2:**
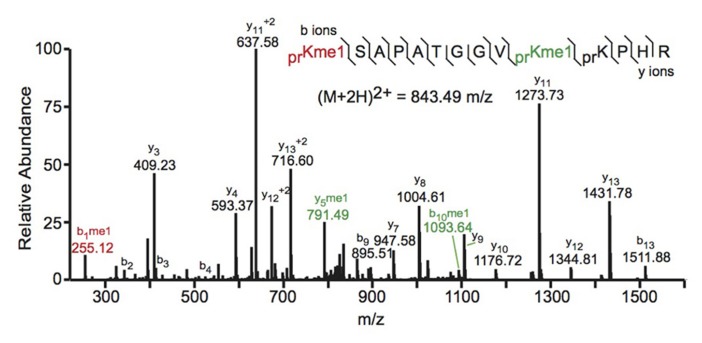
**Bottom-up collision induced dissociation mass spectrum of the (M+2H)^2^+^ H3 27–40 peptide containing K27me1 and K36me1**. The sequence and mass of the precursor peptide ion are denoted in the figure. The lines between the amino acids indicate observed b ions (N-terminal) and y ions (C-terminal). Fragment ions *m*/*z* as well as the precursor m/z were measured in a linear ion trap. The fragment ion highlighted in red was used to identify the K27me1 modification, and the fragment ions highlighted in green were used to assign the K36me1 modification. For example, the mass difference between the y4 and y5 ions is 198.12 Da, which corresponds to the mass of a propionylated and mono-methylated lysine residue, indicating that lysine 36 is monomethylated. Spectra is from data obtained in Zee et al. (2010).

Bottom-up MS commonly employs collision-induced dissociation (CID) fragmentation in which the peptide bond is cleaved upon collision with inert gas. An example of a spectrum obtained using CID is shown in **Figure 2**. The Allis and Hunt labs jointly demonstrated the utility of bottom-up MS in identifying novel PTMs. Specifically, the groups demonstrated that H4 can be monomethylated at position 3 by the methyltransferase PRMT1 (Strahl et al., 2001). Subsequent studies also identified the core PTM H3K79 methylation (Ng et al., 2002; van Leeuwen et al., 2002). Despite these and many other successful histone analyses with CID fragmentation, there are some drawbacks to using this method. Firstly, histone tails contain many basic residues that prevent random protonation and, therefore, promote non-random cleavage of the peptide backbone. Non-random backbone cleavage can lead to incomplete sequence coverage and reduced confidence in assigned sequences. Furthermore, labile PTMs, such as phosphorylation, tend to be lost with CID, preventing localization of these marks to a specific residue.

Recently, bottom up MS and other methods have uncovered novel histone PTMs. For example, bottom-up MS has identified locations on H2A, H2B, and H4 that bear β-N-acetylglucosamine (OGlcNAc). These modifications occur on specific serine and threonine residues and may lead to transcriptional repression as indicated by increased DNA condensation in cells containing increased levels of OGlcNAc (Sakabe et al., 2010). A later study by the Zhao group employed mutational analysis and immunobiology to demonstrate that H3S10 can be OGlcNAcylated. The study also revealed that total histone OGlcNAcylation varies throughout the cell cycle, indicating that this modification may modulate chromatin function during cell cycle progression (Zhang et al., 2011). Despite these important advances, the role of OGlcNAcylation remains poorly understood. The Zhao group also demonstrated that specific lysines are propionylated or butyrylated by acetyltransferase enzymes (Chen et al., 2007; Zhang et al., 2009). Another novel histone PTM, lysine crotonylation (Kcr), was recently discovered using bottom-up MS. Kcr sites were discovered on 28 lysine residues from all four core histones and H1. This modification associates with active chromatin and is enriched at actively transcribed regions of male germinal post-meiotic cells (Tan et al., 2011). Recently, a combination of MS and biochemical techniques were used to discover a glutathionylation site on H3 that causes chromatin to adopt a more open conformation (García-Giménez et al., 2013). Similarly, lysine malonylation and succinylation sites have been identified, all of which are contained in the nucleosome core region (Xie et al., 2012). The functional impact of these modifications are largely uncharacterized and further study is needed to determine their roles in chromatin biology.

### MIDDLE-DOWN AND TOP-DOWN MASS SPECTROMETRY

In order to study the co-occurrence of two or more PTMs by MS, they must be contained on the same peptide. Bottom-up MS is therefore limited in this approach because the tryptic peptides are small and, thus, will not contain all of the PTM sites. Middle-down and top-down MS involves the analysis of larger peptides or entire proteins, respectively, and are therefore more useful in studying combinatorial histone codes (see **Table 1** for a summary of the advantages and disadvantages of each MS approach). To obtain larger protein fragments for middle-down MS that contain many histone PTM sites, a protease other than trypsin must be used. Generally, a protease that cleaves after a single residue is ideal to reduce missed-cleavage events. Endoproteinase AspN is commonly used to generate the H4 1–24 peptide, which contains all known PTM sites, and endoproteinase GluC is often used to generate the H3 1–50 peptide, which contains most of the known PTM sites.

**FIGURE 3:**
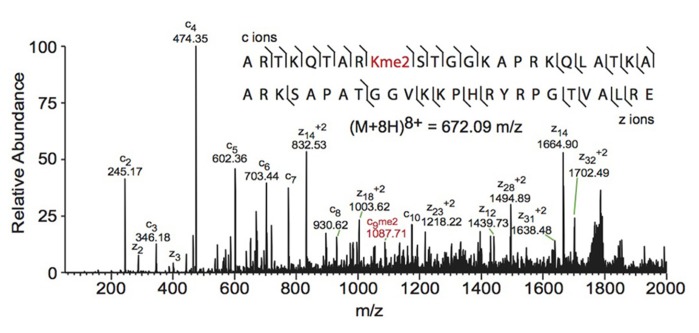
**Middle-down electron transfer dissociation mass spectrum of the (M+8H)^8+^ H3 1–50 peptide containing K9me2**. The sequence and mass of the precursor peptide ion are denoted in the figure. The lines between the amino acids indicate observed c ions (N-terminal) and z ions (C-terminal). Fragment ions *m*/*z* as well as the precursor *m*/*z* were measured in a linear ion trap. The fragment ion highlighted in red was used to assign the dimethyl group to lysine 9. Spectra is from data obtained in Young et al. (2009).

Electron capture dissociation (ECD) and electron transfer dissociation (ETD) fragmentation methods are often used for middle- and top-down MS analysis of histones. In both methods, an electron reacts with the positively charged peptides to produce a radical which then induces fast cleavage at the N-Cα bond of the peptide backbone. In ECD, low energy electrons are used to produce the radicals in the ion cyclotron resonance cell of a Fourier transform mass spectrometer. ECD spectra are obtained by averaging data over many scans, a time consuming process compared to other fragmentation methods. As such, ECD is not compatible with the timescale needed for liquid chromatography (Syka et al., 2004; Udeshi et al., 2007; Wiesner et al., 2008). On the other hand, ETD, developed by the Hunt lab, uses an anion carrier to deliver electrons. Fragmentation by ETD is very efficient and does not require averaging of data to generate spectra and is therefore amenable to the liquid chromatography timescale (Syka et al., 2004; Udeshi et al., 2007). Furthermore, the use of an anion carrier allows ETD to be implemented in many instrument types, increasing its versatility compared to ECD, which can only be done in Fourier transform mass spectrometers (one of the most expensive instruments). The mechanism of fragmentation for both ECD and ETD is not biased towards any given amino acids, allowing for more even backbone cleavage compared to CID. Even cleavage along the backbone generates more fragmentation ions and consequently allows for better sequence coverage and confidence in assigned sequences (Udeshi et al., 2007; Wiesner et al., 2008). ETD and ECD are better suited to preserve labile PTMs than CID, thereby increasing the utility of these methods for PTM analysis. Histone peptides generated for middle-down MS are highly positively charged because they contain a large number of arginine and lysine residues. ECD and ETD fragmentation require high charge states, and so these peptides are highly amenable to these fragmentation methods. Conversely, small tryptic peptides seldom achieve high charge states, and consequently CID is more commonly used in bottom-up histone analysis (Wiesner et al., 2008). An example of a spectrum obtained using ETD is shown in **Figure 3**.

Middle-down analysis of histone tail peptides requires different chromatography than bottom-up peptides. Since middle-down histone tail peptides are more highly charged, they do not bind well to the hydrophobic stationary phase used in RP chromatography and typically elute in the void volume or experience poor separation. Weak cation exchange (WCX) hydrophilic interaction liquid chromatography (HILIC) utilizes a hydrophilic stationary phase coupled to an organic mobile phase and has been a successful alternative chromatographic method for separating differentially modified tail peptides. HILIC was originally developed as an off-line prefractionation method due to the high salt composition of the buffers needed for WCX. Each fraction requires further purification and separate MS analysis. The Kelleher group has extensively used this method for histone analysis. For example, the group employed off-line WCX-HILIC to discover 150 differentially modified forms of H3.2 in asynchronous and butyrate-treated cells (Garcia et al., 2007b). The group also used off-line WCX-HILIC coupled with top-down MS to identify 42 unique combinatorial codes on H4 in HeLa cells (Pesavento et al., 2008). HILIC was further adapted in 2008 to allow for on-line separation by the addition of a pH gradient that removed the salt requirement, drastically reducing sample and time requirements (Young et al., 2009). Direct infusion is typically used for top-down analysis of histones because differentially modified forms are difficult to separate by chromatography (Zee et al., 2011), although some chromatographic methods have proven useful. Notably, Tian et al. (2012) implemented 2D liquid chromatography using RPLC in the first dimension and saltless WCX-HILIC in the second dimension to characterize PTMs of intact histones. Using only 7.5 μg of purified core histones, the authors were able to identify 708 unique histone isoforms, an improvement over other 1D chromatographic platforms (Tian et al., 2012).

One caveat of middle- and top-down MS is decreased sensitivity relative to bottom-up MS. Larger peptides can occupy a larger distribution of charge states, resulting in a dilution of signal for any given charge state compared to smaller peptides. Furthermore, the greater number of PTM sites on middle-down peptides increases the number of possible modified forms, which dilutes the signal for any given combination of PTMs. Dilution of signal is greatest for top-down MS because the intact protein has the maximal number of potential modified forms and charge states. Thus, sensitivity is sacrificed for greater connectivity in top- and middle-down experiments. **Table 1** summarizes the relative advantages and disadvantages of each MS approach.

### HISTONE VARIANTS

H1, H2A, H2B, and H3 each have different gene variants that can be incorporated into nucleosomes to influence chromatin structure. These non-canonical histones are central to many vital nuclear processes such as chromosome segregation, DNA repair, and sex chromosome condensation (Talbert and Henikoff, 2010). MS has been employed to distinguish these histone variants, some of which differ by only a single amino acid. Bottom-up MS can successfully be used for this analysis, but middle-down or top-down analysis is ideal when identifying small variations in sequence and differences in combinatorial PTMs between variants.

Recent bottom-up MS experiments have identified and characterized histone variants. Two novel primate-specific H3 variants, H3.X and H3.Y, were identified using bottom-up MS. Depletion of H3.Y results in the down-regulation of cell cycle-associated and chromatin structure regulatory genes and a concomitant decrease in cell growth, indicating that this variant is likely involved in regulating cell proliferation (Wiedemann et al., 2010). Another recent study used bottom-up MS to characterize single and combinatorial PTMs on H3.2 and H3.3 in mouse embryonic stem cells lacking Suz12, a member of the polycomb repressive complex 2 that is required for cell differentiation. Using both CID and ETD, the authors were able to identify peptides from H3.2 and H3.3, and localize a total of 46 modifications to 22 different locations within these peptides. Results indicated that Suz12-deficient cells experience a dramatic reduction of H3K27me2 and H3K27me3 and an increase in H3K27ac, highlighting this residue as an acetyl/methyl switch. H3K27ac was accompanied by a corresponding increase in H3K36ac, a combination that had not been previously described in mammalian embryonic stem cells (Jung et al., 2010).

Top-down MS analysis has been used to thoroughly characterize all canonical histones and a vast majority of their known variants. Notably, the Kelleher group has published a series of reports on top-down MS analysis of H2A and H2B variants. These studies revealed that most variants remain unmodified and do not vary in expression levels throughout the cell cycle (Boyne et al., 2006; Siuti et al., 2006; Thomas et al., 2006). Middle- and top-down analyses of H3 variants revealed very minor differences in PTM levels and patterns compared to canonical H3 (Thomas et al., 2006; Garcia et al., 2008).

### QUANTIFICATION

Relative and absolute quantification of histone PTMs and variants is necessary when comparing two or more sets of experimental data. Although MS is inherently quantitative for mass measurements, differences in ionization efficiencies between peptides do not allow simple quantitative comparisons. In spite of this, there are now several methods available to extend MS capability to include quantitation comparisons between different peptides (Eberl et al., 2011). Several of these methods allow for several samples to be combined and run simultaneously (multiplexing), which vastly decreases the time required for MS analysis. These methods can be broadly characterized into three main groups: label-free, chemical tags, and metabolic labeling.

Perhaps the simplest method to compare peptide or PTM abundances is label-free quantitation. The relative abundance of a particular modified peptide form can be obtained by integrating the area under the peak for each charge state and dividing it by the total area for that peptide in all of its modified forms, obtaining a fractional occupancy. For this method to be effective, however, the sample must contain a homogenous pool of peptide fragments, which can be accomplished through the use of chemical derivatization or a highly specific protease as described earlier.

Labeling peptides with chemical tags also allows for quantitation of peptide abundance level changes between samples and is amenable to multiplexing. Generally this method involves addition of different stable chemical tags to samples from different backgrounds. This differential labeling creates a mass difference between the samples that can be measured via MS to determine the origin of each peptide. One of the first examples of this technique involved converting carboxylic acid moieties to their respective ethyl esters using either deuterated or non-deuterated ethanolic HCl. Syka et al. (2004) used this method to differentially label asynchronous and mitotic histone peptides to quantify differences in modification state between the two populations. Results indicated that histones in cells undergoing mitosis have a greater level of phosphorylation compared to asynchronous cells (Syka et al., 2004). A similar approach involves derivatization of two different samples with d_0_- or d_5_-propionic anhydride, which, as mentioned earlier, propionylates unmodified lysines. This technique was used in combination with bottom-up MS to compare H3 and H4 variants and PTM profiles between wild type and G9a methyltransferase knock-out (KO) cells. Total histones were treated with d_0_-propionic anhydride followed by tryptic digestion. A second derivatization was completed to modify the newly exposed N-terminus using d_0_-propionic anhydride for wild type cells or d_5_-propionic anhydride for KO cells (Δm = +5 Da). Results indicated that the KO cells experienced little differences in H4 PTM profiles but experienced significant differences in H3 PTM profiles, such as a notable decrease in H3K9me and an increase in H3K14ac (Plazas-Mayorca et al., 2009, 2010). This method was also used to detect differences in histone PTM levels between wild-type IMR90 cells and Ras-induced senescent IMR90 cells. The senescent cells underwent a decrease in H3K9ac, H3K4me2, and H3K4me3 and an increase in H3K9me3, H3K27me3, and K3K4me3, indicating that gene repression is likely involved in this model of senescence (Chicas et al., 2012). Stable isotopic labeling was also used recently to measure the rate of histone acetylation for several marks and measure histone turnover. Briefly, [^13^C]glucose was added to human cells to metabolically label new acetyl groups as well as alanine, allowing new histones to be differentiated from old. Results indicated that specific acetylation turnover rates vary depending on location and neighboring modifications and also vary between cells in different biological conditions. The authors also found that newly synthesized histones accumulate modifications more slowly than pre-existing histones (Evertts et al., 2013).

One of the most common metabolic labeling techniques for histone PTM quantification is stable isotope labeling by amino acids in cell culture (SILAC), where selected amino acids in cell culture media are replaced with isotopically labeled amino acids to label all proteins containing that amino acid (Ong et al., 2002). This method was used to thoroughly investigate PTM profiles of the four core histones during various stages of the cell cycle (Bonenfant et al., 2007). SILAC was also used to quantitate differences in H3 and H4 PTMs both singly and in combination in four breast cancer cell lines compared to normal breast cells. Results demonstrated that several marks experience a significant difference in PTM abundance between cancer cells and normal cells, indicating that these modifications are important in the pathology of breast cancer (Cuomo et al., 2011). Another variation of SILAC, termed “heavy methyl” SILAC can be used to isotopically label methyl groups by adding (^13^CD_3_)-*S*-adenosyl methionine (SAM), the methyl donor used by all methyltransferases, to cell media lacking methionine. Modification by this labeled cofactor imparts a 4 Da mass shift that can easily be detected by MS. This approach is highlighted in **Figure 4**, where the mass spectra of unmodified and heavy-methyl labeled H3 27–40 peptide (+2) containing K27me1 from HeLa cells after 12 h of incubation with heavy SAM is shown. Heavy methyl SILAC has been used to quantify “new” versus “old” methylation in cells and to determine the turnover of methylated peptides at different cell stages (Ong et al., 2004; Fodor et al., 2006; Zee et al., 2010). SILAC is highly amenable to multiplexing because different groups of cells can be labeled with different isotopically labeled amino acids, allowing for the simultaneous analysis of many samples.

**FIGURE:**
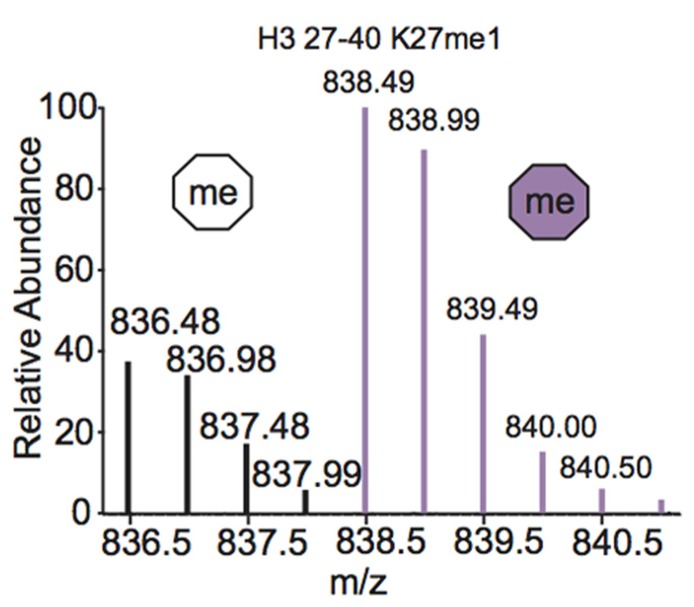
**Mass spectrum displaying the (M+2H)^2+^ H3 27–40 peptide containing K27me1 from cells 12 h after addition of (^13^CD_3_)-*S*-adenosyl methionine**. Both unlabeled peptide (white hexagon) and heavy-methyl labeled peptide (purple hexagon) are observed. Spectra is from data obtained in Zee et al. (2010).

## METHODS FOR PROBING THE BIOLOGICAL RELEVANCE OF IDENTIFIED HISTONE COMBINATORIAL CODES

The first half of this review focused on using MS to confirm, identify, and quantify histone variants and combinatorial PTMs. As new PTM codes are identified by MS, questions arise about the biological significance of these marks. A vast array of studies can be implemented to profile the biological importance of discovered combinatorial PTM patterns, using techniques from biochemistry, biophysics, chemical biology, and others. This section will highlight recent developments designed to further probe the biological role of histone combinatorial codes.

### READER PROTEINS ARE RESPONSIBLE FOR THE BIOLOGICAL READOUT OF HISTONE MODIFICATIONS

Histone PTMs can directly alter chromatin structure to regulate nuclear processes such as transcription. For example, ubiquitylation of H2B, a mark associated with transcription elongation (Fleming et al., 2008), is believed to physically separate chromatin fibers, allowing access to underlying DNA. In support of this hypothesis, a recent study employed fluorescence anisotropy to measure internucleosomal distances of chromatin fibers containing uniformly ubiquitiylated H2B to reveal that this modification results in a decrease in chromatin compaction. Compaction of chromatin fibers is likely blocked by the steric bulk of the ubiquitin moiety (Fierz et al., 2011).

**FIGURE 5:**
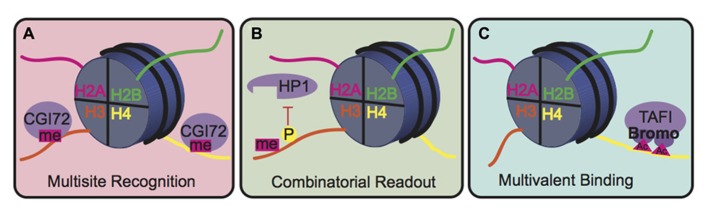
**Binding modes for histone readers. (A)** In multisite recognition, a reader can recognize the same mark in several locations, either on the same tail or on different tails. **(B)** Combinatorial readout occurs when a reader recognizes several modifications simultaneously. **(C)** Multivalent binding occurs when several reader domains within a protein complex engage several marks simultaneously.

The biological consequence of many PTMs, however, is mediated by the action of reader proteins that bind to these sites. The binding modes of reader proteins can be characterized into three main groups: multisite recognition, combinatorial readout, and multivalent binding (Fierz and Muir, 2012; **Figure 5**). Multisite recognition occurs when readers can bind to several different marks. For example, CGI-72, a member of the MBT domain family, can bind H3K4me1 and H4K20me1 (Kim et al., 2006). Similar binding flexibility has been observed in many other reader domain families, including bromodomains, plant homeo-domain fingers and royal superfamily domains (Fierz and Muir, 2012). Many reader domains have low binding affinities with their respective PTMs, which could explain the observed binding plasticity (Fierz and Muir, 2012). A single reader may also engage histone peptides by interacting with several modification sites simultaneously, a binding mode termed “combinatorial PTM readout.” Neighboring PTMs may enhance or impede reader binding. For example, a single bromodomain of Brdt, the mouse homolog of TATA-binding protein-associated factor-1 (TAF1), can simultaneously bind two acetyl-lysine residues on H4 (Morinière et al., 2009). As an opposing example, H3S10phos prevents binding of heterochromatin protein 1 (HP1) to the adjacent H3K9me3 (Fischle et al., 2005). In multivalent binding, protein complexes containing multiple reader domains can bind several coincident PTMs (Ruthenburg et al., 2007; Wang and Patel, 2011; Fierz and Muir, 2012). For example, TAF1, a component of the TFIID transcription factor, contains two bromodomains that bind to acetylated lysines on H4 to localize this transcription factor to transcriptionally active genes (Jacobson et al., 2000). This multivalent binding allows for the engagement of several PTMs simultaneously, thereby enhancing specificity and affinity of the reader complex compared to isolated domains. For example, the binding affinity of the double-bromodomain of TAF1 is 7–27 fold greater when bound to multiple acetyl-lysine residues compared to a single modification (Wang and Patel, 2011).

### TECHNIQUES TO GENERATE A HOMOGENOUSLY MODIFIED POOL OF HISTONES

Studies aimed to characterize the specific role of combinatorial or isolated PTMs are greatly enhanced by methods that generate pools of chemically defined histones. Since histones from different parts of the genome contain vastly different PTM profiles, these homogenous pools cannot easily be obtained from cells. Fortunately, several techniques have been developed to create pools of chemically defined histones, nucleosomes, and chromatin to facilitate studies aimed to identify the biological relevance of specific marks.

Synthetic peptide strategies can be employed to generate small peptides of defined composition. In these methods, amino acids are added sequentially to a growing polypeptide chain to produce a highly purified pool of peptides with identical chemical composition. Modified amino acids, such as N-∊-acetyl-lysine, can also be easily incorporated. One of the first experiments to use this methodology was completed in 1974, where the H4 15–21 peptide containing ^14^C-aceylated Lys16 was synthesized to test the substrate specificity of purified histone deacetylases (HDACs). Results demonstrated that synthetic H4 15–21, but not the chymotryptic-digestion dervived H4 1–37, is deacetylated, indicating that there is a minimal sequence requirement for HDAC function (Krieger et al., 1974). Synthetic peptides have also been used to identify readers of specific marks or combinations of marks, as described later. Despite their utility, one critical limitation of synthetic peptide strategies is that only small peptides (usually under 50 amino acids) can be generated due to decreasing efficiency of peptide bond formation as length increases. This method is therefore not conducive to studies aimed to characterize the effects of PTMs on the whole-protein level or higher.

Native chemical ligation (NCL) and expressed protein ligation (EPL) can be used to circumvent the length limit inherent to synthetic peptide synthesis. NCL is a chemical strategy that combines two synthetic peptides with a native amide bond (Dawson et al., 1994). While this is useful to generate longer peptides than synthetic peptide synthesis alone, the length of NCL-generated peptides is still limited to the length of the component synthetic peptides. Therefore, several successive rounds of NCL are required to produce long peptides or whole proteins. A recent study employed sequential NCL to produce full-length H3 containing acetylated K56, a mark involved in DNA transcription, replication, and repair. The authors were able to use this synthetic histone to show that H3K56ac increases binding of a DNA-associated enzyme, LexA, about 3-fold over histones lacking this PTM (Shimko et al., 2011). However, sequential NCL can become time consuming because each peptide segment must be manufactured separately by synthetic means. EPL, on the other hand, involves ligation of a synthetic peptide to a recombinant protein, thereby allowing incorporation of specific modifications on intact proteins without needing to synthesize the entire protein (Muir et al., 1998). Manohar et al. (2009) used EPL to generate H3 containing acetylated K115 and/or acetylated K122, which are located at the nucleosomal dyad. Nucleosomes were reconstituted from these semi-synthetic H3 histones, and the rates of thermal repositioning along DNA were measured. Repositioning was enhanced in the acetylated H3 isoforms, highlighting the importance of acetylation at the dyad in mediating nucleosome sliding (Manohar et al., 2009). One major drawback of EPL is that the modification of interest must be located near one of the protein termini (usually < 50 resides) due to the length limit in generating the synthetic peptide (Fierz and Muir, 2012).

Non-sense-suppression mutagenesis techniques can be used to incorporate a modified amino acid at any position in proteins in order to study the biological relevance of the modification (Davis and Chin, 2012). The Schultz group demonstrated the utility of this method for histone PTM analysis by evolving an orthogonal mutant *Methanococcus jannaschii* tyrosyl amber suppressor transfer RNA (tRNA) and Tyr *Mj*tRNA CUR/tyrosyl-tRNA synthetase (MjTyrRS) pair. This pair successfully incorporates (Se)-phenylselenocysteine (PhSeCys) at the amber TAG codon in *Escherichia coli*. PhSeCys can then be converted to N-acetyl-lysine, serine phosphorylation, and mono-, di-, and tri- N-methyl-lysine (Guo et al., 2008; Wang et al., 2012). Similarly, the Chin group evolved a pyrrolysyl tRNA synthetase and tRNA pair from *Methanosarcina barkeri* to incorporate N-∊-acetyl-lysine and N-∊-methyl-lysine at TAG codons (Neumann et al., 2009; Nguyen et al., 2009). The Chin group used this method to generate H3 containing acetylated K56 that were then reconstituted into nucleosomes and nucleosomal arrays to study the effect of this modification on chromatin compaction, remodeling, and DNA breathing. Results indicated that the modification does not significantly affect chromatin compaction, as measured by sedimentation velocity analysis, or remodeling, as demonstrated by the lack of recruitment of remodelers to this site. However, the modification did increase DNA unwrapping about 7-fold compared to nucleosomes lacking this modification (Neumann et al., 2009). Kim et al. (2012) expanded on this system to evolve a synthetase/tRNA pair capable of inserting the newly discovered modification, ∊-N-crotonyl-lysine, into histones. Further development of this methodology led to the evolution of more orthogonal synthetase/tRNA pairs to incorporate ∊-N-propionyl-, ∊-N-butyryl-, and ∊-N-crotonyl-lysine in histones (Gattner et al., 2013). Nonsense-suppression mutagenesis cannot be accomplished for large modifications, such as ubiquitylation, because the tRNA/synthetase pairs cannot accommodate bulky substituents. Several alternative methods have been developed to incorporate ubiquitin as described elsewhere (Chatterjee and Muir, 2010).

In addition to the above-mentioned methods to generate homogenously modified peptides or protein, techniques exploiting cysteine chemistry can be used to generate peptides containing modified lysine analogs at specific residues. Cysteine is the only amino acid that contains a highly nucleophilic sulfhydryl group and therefore has unique chemical reactivity. The Shokat group demonstrated that cysteine can be converted into methyl-lysine analogs (MLAs) by alkylating the sulfhydryl groups with electrophilic mono-, di-, and tri-ethylamines. This method can therefore be used to easily incorporate MLAs into any location within the protein by mutating the amino acid of interest to a cysteine (Simon et al., 2007). This technique was later expanded to produce acetylated lysine analogs (Li et al., 2011). One drawback, however, is that synthesis of histones containing modifications at several sites becomes very challenging (Fierz and Muir, 2012). Recently, Yuan et al. (2011) used this MLA approach to generate H3K36me2 and H3K36me3 analogs to investigate the mechanism of PRC2, a methyltransferase responsible for trimethylating H3K27. PRC2 activity was measured on histones containing the H3K36 MLAs, and results indicated that PRC2-mediated trimethylation of H3K27 was completely inhibited, highlighting H3K36me as a regulator of PRC2 function (Yuan et al., 2011).

### IDENTIFYING READERS AND DETERMINING THEIR SPECIFICITY FOR A SPECIFIC COMBINATORIAL CODE

Readers bind to specific combinatorial codes to initiate biological responses. For this reason, many studies aim to identify the readers of particular PTM combinations and determine the specificity of these readers for the given code.

Affinity purification techniques have proven useful in identifying specific binders of a combinatorial code of interest. One of the first of these methods uses a chemically defined substrate peptide to pull down interacting proteins. For example, the Allis group used biotin-tagged synthetic H3 1–20 peptides containing di- or tri-methylated K4 to pull down readers from cell lysate. Results indicated that WDR5, a member of MLL1, MLL2, and hSet1 methyltransferase complexes, binds to di- and tri- methylated H4 (Wysocka et al., 2005). One drawback of this method, however, is the lack of quantitation and inability to differentiate specific binders from non-specific binders. To address this issue, many techniques have supplemented affinity purification methods with SILAC. One such method, developed by Vermeulen et al. (2007) involves using SILAC in combination with a pull-down approach using histone tails as bait. In this study, the H3 1–17 peptide was synthesized containing a biotin tag and either unmodified or trimethylated K4. The unmodified and modified peptides were incubated with “light” or “heavy” cell extracts, respectively, followed by a biotin pull-down and MS analysis. H3K4me3-specific readers were determined by comparing identified binding proteins in the unmodified peptide pool with those in the modified peptide pool. Results indicated that several subunits of the transcription factor complex, TFIID, directly bind H3K4me3 (Vermeulen et al., 2007). The group later used this method to identify readers of repressive marks H3K9me3, H3K27me3, and H4K20me3 and activating marks H3K4me3 and H3K36me3 (Vermeulen et al., 2010). One caveat of using histone peptides as bait is that readers may interact with other portions of the nucleosome or chromatin and may therefore be missed in pull-downs. To avoid this issue, other studies have used higher order structures to identify readers in a more biological context. For example, Bartke et al. (2010) developed and employed SILAC nucleosome affinity purification (SNAP) to identify readers that bind a combination of H3 lysine methylation and CpG-methylated DNA within intact nucleosomes (Bartke et al., 2010). The Fischle group developed an affinity purification protocol paired with SILAC to characterize the interactome of H3K4me3 and H3K9me3 in homogenously modified chromatin (Nikolov et al., 2011). These pull-down approaches can be supplemented by chemically cross-linking histones to their readers. To this end, the Kapoor group developed an approach called cross-linking-assisted and SILAC-based protein identification (CLAPSI), in which a photo-crosslinker is engineered into an H3 tail peptide containing H3K4me3. Upon exposure to UV, all readers, including those that bind weakly, will be covalently crosslinked to the substrate peptide, thereby allowing weak binders to be identified (Li et al., 2012).

Peptide and reader domain microarrays have also been employed to identify readers of specific histone PTMs. The Bedford group developed a chromatin-associated domain array (CADOR) chip containing histone-binding domains from the bromo, chromo, tudor, PhD, SANT, SWIRM, MBT, CW, and PWWP families. Fluorescently labeled H3 and H4 peptides containing varying degrees and sites of lysine methylation were flowed over the CADOR chips, revealing novel interactions between readers and modified histones (Kim et al., 2006). Recently, this technique was used to identify the transcriptional coactivator TDRD3 as a reader of H3R17me2a and H4R3me2a (Yang et al., 2010).

Peptide libraries can also be employed to determine the specificity of a given reader for its histone substrates. To this end, Garske et al. (2008) generated a library of the H4 1–21 tail peptides containing every combination of most PTMs known at the time synthesized to beads. The library was incubated with the double tudor domain of the histone demethylase hJMJD2AA to determine its substrate specificity. A colorimetric assay was used to identify successful binding, followed by MS analysis to identify and determine the relative amounts of bound peptide substrates (Garske et al., 2008). The group later developed an H3 tail peptide library to probe the combinatorial substrate specificity of six chromatin-binding domains (Garske et al., 2010). As an alternate method, the Mrksich group recently measured the activity of histone deacetylase 8 (HDAC8) on differentially acetylated H4 tail peptides using a SAMDI (self-assembled monolayers for matrix assisted laser desorption/ionization time-of-flight MS) assay. Results indicated that local and distal H4 tail sequence mediates HDAC8 activity (Gurard-Levin and Mrksich, 2008). The group later used the SAMDI assay to identify isoform-specific substrates for four different lysine deacetylases (KDACs) in order to profile activity of these enzymes throughout the cell cycle (Gurard-Levin et al., 2010).

### STRUCTURAL CHARACTERIZATION OF HISTONES

Many techniques have been employed to study the structure of nucleosomes in various contexts to understand the biophysical basis of their genetic function. X-ray crystallography and nuclear magnetic resonance (NMR) spectroscopy have proven useful in elucidating the structure of histone proteins in various contexts, which have consequently revealed important aspects of nucleosome function and interaction with other proteins.

In X-ray crystallography of nucleosomes a beam of X-rays is focused on a solidified protein/DNA crystal, which then diffracts these beams. The 3-dimensional structure of the crystallized nucleosome can then be determined at atomic resolution from a series of diffraction patterns. The crystal structure of the nucleosome containing two copies of each canonical histone protein was solved to 2.8 Å resolution in 1997 by the Richmond group (Luger et al., 1997). Since that time, X-ray crystallography has been used to determine the structures of nucleosomes containing non-canonical histone variants. For example, the Kurumizaka group recently solved the structure of nucleosomes containing H3.2 and H3.3, demonstrating that the structures are near matches to that of the canonical H3.1-containing nucleosome. The residues that differ between these variants are located on the nucleosomal surface, suggesting that these variants may affect chromatin biology by altering interactions with other proteins rather than imparting structural changes (Tachiwana et al., 2011a). The structure of the nucleosome containing centromere protein A (CENP-A), the H3 variant found at centromeric chromatin, has also been solved using X-ray crystallography (Tachiwana et al., 2011b), confirming some structural differences compared to canonical nucleosomes, including a bulged loop L1 that is critical for centromere targeting and had been identified by a crystal structure of the sub-nucleosomal (CENP-A/H4)_2_ heterotetramer (Sekulic et al., 2010). In addition, the 13 bp from both ends of the nucleosomal DNA are unstructured in the CENP-A nucleosome crystal structure in contrast to corresponding ones containing canonical nucleosomes (Luger et al., 1997; Tachiwana et al., 2011b). This latter finding is consistent with the DNA unwrapping observed on CENP-A nucleosomes at endogenous centromeres (Hasson et al., 2013).

X-ray crystallography and NMR have also been extensively used to determine the structure of histones bound to chaperones and readers to provide insight into the mechanism of association. As an example of a histone-chaperone complex, the structure of the H3.3-specific chaperone death domain-associated protein (DAXX) in complex with H3.3–H4 heterodimer was solved (Elsässer et al., 2012; Liu et al., 2012). Based on these structures, Elsässer et al. (2012) provides an explanation for the specificity of DAXX for H3.3, which differs from H3.1 to H3.2 by only five residues. The structure suggests that H3.3 G90 (corresponding to M90 in H3.1 and H3.2) interacts with DAXX through a series of water-mediated hydrogen bonds. The crystal structure of DAXX in complex with the H3.3(G90M)/H4 heterodimer indicates that this hydrogen bond network is disrupted, providing a structural explanation for DAXX’s preference for G90 in H3.3 over M90 in H3.1 and H3.2 (Elsässer et al., 2012). As another example, the structure of the chaperone FACT (facilitates chromatin transcription) in complex with the H2A/H2B heterodimer was also solved recently, demonstrating that the U-turn motif of FACT is responsible for binding to the α-1 helix of H2B (Hondele et al., 2013). X-ray crystallography and NMR have also been extensively used to determine the structures of readers bound to substrate histones and histone peptides. For example, herculean and cool methyl-TROSY experiments have revealed the binding mechanism of the high mobility group nucleosomal protein 2 (HMGN2) to nucleosomes. HMGN2 binds to an acidic patch in the H2A/H2B dimer as well as DNA near the entry/exit site, effectively securing the nucleosome in a specific position on the DNA. The experiments also demonstrate that HMGN2 binding prevents H1 interaction with the nucleosome (Kato et al., 2011). As another example, the structure of the reader UHRF1 (ubiquitin-like, containing plant homeodomain and really interesting new gene finger domains 1) in complex with the tail of H3 containing unmodified R2 and methylated K9 has been solved using X-ray crystallography. UHRF1 contains two reader domains linked by a 17 aa linker. The linker forms a binding pocket that binds the H3 peptide and forces it into a compact helical conformation which then orients the tail in the required register for the double reader domains to bind R2 and methylated K9. Isothermal titration calorimetry (ITC) and NMR experiments also revealed that phosphorylation of UHRF1 at S298 inhibits interaction with H3 (Arita et al., 2012). Many other structures of readers in complex with substrate proteins have been obtained, revealing overarching themes in the molecular mechanisms for readout of specific PTMs. For example, mono- and di-methyllysine reader domains tend to contain a binding pocket with 2 to 4 aromatic residues as well as an aspartate residue that hydrogen bonds to the methylammonium proton of the methyllysine. This hydrogen bond cannot be formed when the lysine is tri-methylated, allowing for exclusion of this PTM in the binding pocket (Taverna et al., 2007). Please refer to some recent reviews for more detailed reader binding mechanisms for a variety of marks (Taverna et al., 2007; Musselman et al., 2012).

### DYNAMIC CHARACTERIZATION OF HISTONES

Many studies have investigated the dynamic properties of histones in order to better understand the biophysical basis of histone function in multiple biological contexts. A wide range of techniques, at both ensemble and single molecule levels, have shed insight into the dynamic properties of histones, nucleosomes, and nucleosome-protein complexes. These techniques, especially when combined with structural information, have revealed important aspects of nucleosome function.

Hydrogen-deuterium exchange (HDX) is a useful technique to measure protein dynamics (Englander, 2006). In this method, proteins or protein/nucleic acid complexes are solvated in deuterium oxide (heavy water) over a course of time. Amide hydrogens on the peptide backbone readily exchange with solvent to become deuterated unless they are participating in a hydrogen bond. Therefore, in stably folded proteins, amide hydrogens involved in forming secondary structure are protected from solvent while amide hydrogens that are not involved in hydrogen bonds, such as flexible loops, will readily exchange. However, proteins do not exist as rigid bodies in solution, and transient unfolding events in secondary structures briefly break hydrogen bonds, exposing amide protons for exchange with deuterons from the heavy water. More stable secondary structures will undergo fewer transient unfolding events relative to less stable secondary structures and will therefore have slower exchange rates. Deuterium incorporation, as measured by NMR or MS, can therefore be used to gage stability of protein structures. HDX can also be used to map binding interfaces as amino acids involved in hydrogen bonds to other proteins will be protected from exchange (Englander, 2006).

Hydrogen-deuterium exchange has been employed to study the dynamics of histone proteins in many contexts, including tetramers, nucleosomes, nucleosomal arrays, and protein complexes. For example, the Cleveland group used HDX coupled to MS to compare the dynamics of the (CENP-A/H4)_2_ tetramer to the canonical (H3/H4)_2_ tetramer. Regions of the (CENP-A/H4)_2_ tetramer, corresponding to CENP-A residues 94–116 and H4 residues 58–78 and 86–91, experience significantly slower exchange than the corresponding regions of the (H3/H4)_2_ tetramer. These observations indicated that the (CENP-A/H4)_2_ tetramer assumes a more rigid conformation than the canonical tetramer (Black et al., 2004). A later study conducted by the same group used HDX-MS on CENP-A-containing and canonical nucleosomes to demonstrate that both CENP-A/H4 and H3/H4 within nucleosomes are<3 orders of magnitude more rigid compared to their respective heterotetramers. This increased rigidity occurred on all of the histone fold helices of each histone. Furthermore, CENP-A-containing nucleosomes experienced slower exchange than the canonical nucleosome, indicating that nucleosome rigidity is important in defining centromeric chromatin (Black et al., 2007). Dynamics of canonical and CENP-A-containing nucleosomal arrays have also been measured using HDX-MS to determine the effects of this substitution on nucleosomes in chromatin context. Results indicated that the αN helix of CENP-A contacts superhelical DNA termini of the nucleosome and is more flexible than that of H3 in folded arrays, indicating that DNA is more loosely connected to CENP-A relative to H3. The C terminal region of H2A also experiences increased exchange in CENP-A containing arrays (folded and unfolded) compared to canonical nucleosomes, indicating that H2A may adopt different conformations in CENP-A- containing and canonical nucleosomes (Panchenko et al., 2011).

**FIGURE 6:**
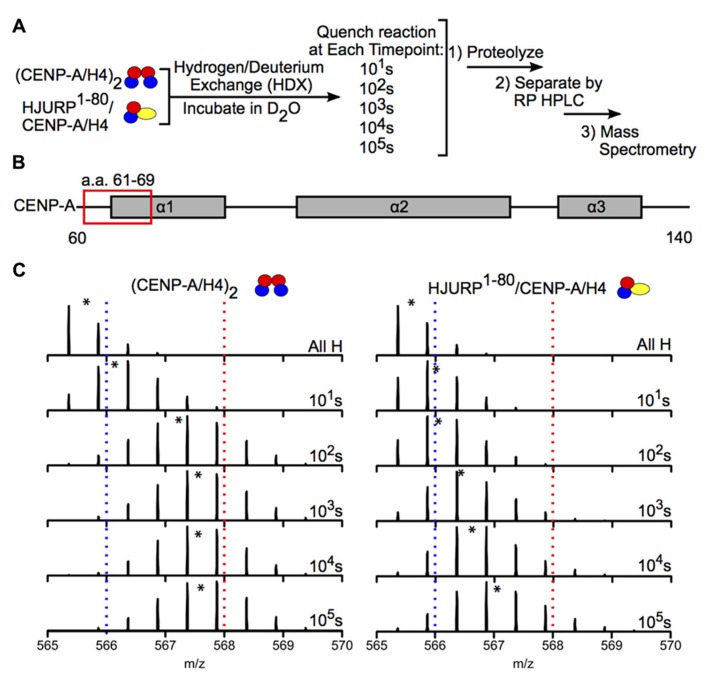
**Hydrogen-deuterium exchange reveals increased stability in CENP-A/H4 dimers bound to HJURP relative to (CENP-A/H4)_2_. (A)** Schematic of experimental setup for HDX experiment. CENP-A/H4 bound to HJURP and (CENP-A/H4)_2_ were incubated in D_2_O to allow for backbone amide exchange. Reactions were quenched after the indicated time points. The protein complexes were proteolyzed, separated via RP-HPLC and analyzed via MS. **(B)** Schematic of CENP-A secondary structural features. The red box indicates the location of a peptide spanning residues 61–69. **(C)** Side-by-side analysis of raw data for CENP-A peptide 61–69 from (CENP-A/H4)_2_ (left) and HJURP^1^^-^^80^/CENP-A/H4 (right). Black asterisks denote centroid locations, and dotted red and blue lines serve as guideposts to highlight the differences in *m*/*z* shifts between the two complexes. Note that the CENP-A peptide is more protected from exchange in the HJURP^1^^-^^80^/CENP-A/H4 complex compared to the heterotetramer as evidenced by a lower level of deuterium incorporation throughout the time course, where it takes >100 times as long to achieve the same level of HDX. Data obtained from Bassett et al. (2012).

Hydrogen-deuterium exchange has also been used to interrogate the interaction between chaperones and histone dimers or tetramers. For example, the Black group used HDX to investigate how binding of the chaperone HJURP to the CENP-A/H4 dimer affects the dynamics of the dimer. HJURP binding conferred stability in most of the histone fold helices of CENP-A and H4 within the dimer compared to (CENP-A/H4)_2_, suggesting that rigidity may be required for deposition of these dimers into nucleosomes (**Figure 6**; Bassett et al., 2012). The group also conducted HDX on a truncated HJURP (1–62), which abolishes the interaction between HJURP and the α1 helix of CENP-A, in complex with the CENP-A/H4 dimer. The dimer experienced a significantly less dramatic stabilization in complex with HJURP^1^^-^^62^ relative to HJURP^1^^-^^80^, indicating that a.a. 63–80 are required to achieve full conformational rigidity (Bassett et al., 2012). The Luger group also used HDX to study the interaction between H2A and H2B and the Nap1 chaperone, and found that H2A/H2B dimers are largely stabilized upon binding to Nap1. The region of Nap1 responsible for interaction with histone was also determined (D’Arcy et al., 2013). As a further example, HDX coupled to NMR has been used to determine the binding interface between (H3/H4)_2_ tetramers and Vps75-Rtt109, a chaperone/acetyltransferase complex. Results indicated that the interaction interface is located on four lysine residues on the H3 tail (one of which is acetylated by Vps75-Rtt109), and an adjacent region of H3 (Su et al., 2011). These examples highlight the power of HDX as a tool to measure histone dynamics and function.

A variety of other techniques have been used to measure the dynamic properties of nucleosomes. Förster resonance energy transfer (FRET) has been used at the bulk and single molecule level to determine structural and dynamic changes of nucleosomes in response to many processes, including post-translational modification (e.g., Gansen et al., 2009; Neumann et al., 2009; Lee et al., 2011; Simon et al., 2011) and incorporation of histone variants (e.g., Park et al., 2004; Hoch et al., 2007), amongst others. Single molecule FRET and single-molecule fluorescence-detected linear dichroism have also been employed recently to study DNA breathing at model replication forks. Results show that DNA breathing at the replication fork is enhanced upon binding of the processive bacteriophage T4-coded helicase-primase to the DNA compared to naked replication fork constructs, suggesting that DNA breathing is involved in the initial binding and function of the helicase-primase (Phelps et al., 2013). Additionally, atomic force microscopy (AFM) has been extensively used to characterize chromatin structure, ranging from entire chromosomes down to dinucleosomes (reviewed by Kalle and Strappe, 2012). For example, high-speed time-lapse AFM was used to demonstrate that nucleosomes undergo transient and spontaneous unfolding and sliding events (Miyagi et al., 2011). Optical and magnetic tweezers have also been employed to investigate histone–DNA interactions, nucleosome disassembly, and the structural properties of higher order chromatin (reviewed by Killian et al., 2012). Magnetic tweezers were used to demonstrate that some PTMs located near the dyad decrease nucleosome stability and promote disassembly (Simon et al., 2007). Recently, a novel high-throughput technique called single chromatin molecule analysis in nanochannels (SCAN) has been described to detect several coincident PTMs on histones and DNA. In this method, modifications of interest are tagged with different fluorophores, and the modification content of a single chromatin molecule can be determined by flowing small volumes through nanochannels while measuring florescence of the passing molecules (Cipriany et al., 2010). SCAN was used to determine the coordination between H3K9me3, H3K27me3, and cytosine methylation (mC) in normal and cancer cells. Results indicated that, in normal cells, mC prevents methylation of K27 but promotes methylation of K9, while in cancer cells mC is required for methylation of K27, indicating that aberrant coordination between silencing marks can lead to disease (Murphy et al., 2013).

## SUMMARY

Nucleosomes are highly dynamic DNA/protein complexes whose activity is mediated by a variety of factors including modification, incorporation of variant histones, and interaction with non-histone proteins. Aberrant modification of nucleosomes as well as mutations in other epigenetic regulators have been implicated a variety of diseases including cancer, neurological disease, and autoimmune disease (Portella and Esteller, 2010). Due to the vital role of nucleosomes in regulating nuclear processes, a great effort has been made by laboratories worldwide to characterize and understand nucleosomal modifications, composition, and behavior in cellular context. MS has proven to be an extremely useful tool to identify and quantify specific PTMs in isolation or in tandem to serve as a starting point to understand how nucleosome modification and composition affect cellular processes. The use of other techniques from chemical biology, biochemistry, biophysics, and others has also contributed enormously to this effort. Despite these great advances, much more work is needed to determine the mechanistic roles of specific PTM profiles in regulating nucleosome behavior.

## Conflict of Interest Statement

The authors declare that the research was conducted in the absence of any commercial or financial relationships that could be construed as a potential conflict of interest.
